# Non-pharmaceutical interventions during COVID-19 in the UK and Spain: a rapid realist review

**DOI:** 10.12688/openreseurope.14566.2

**Published:** 2022-08-26

**Authors:** Pilar Serrano-Gallardo, Ana Manzano, Ray Pawson

**Affiliations:** 1 School of Sociology and Social Policy, University of Leeds, Leeds, LS2 9JT, UK

**Keywords:** COVID-19, non-pharmaceutical interventions, complex systems, counterfactuals, evidence-based policy

## Abstract

The paper is located at the crossroads of two modern intellectual movements. The first, evidence-based policy, seeks to locate vital information that will inform and improve key policy decisions on such matters as population health, social welfare, and human wellbeing. The second, complexity theory, describes the nature of the social world and perceives human action as persistently adaptive and social institutions as incessantly self-transformative. The first assumes that policies and programmes can achieve sufficient control to meet specific and measurable objectives. The second assumes that social actions are sufficiently capricious so that the society never conforms to anyone’s plans – even those of the most powerful. The unparalleled resources committed to control the unprecedented attack of the COVID-19 pandemic are the epitome of complexity. The long struggle to contain the virus thus constitutes an ideal test bed to investigate this paradigmatic split. The paper undertakes this mission - focusing specifically on the effectiveness non-pharmaceutical interventions and examining evidence from the UK and Spain.

## Plain language summary

Modern social life consists of millions of interactions as people conduct the routine business of everyday living. We work, we play, we teach, we learn, we shop, we travel, we marry, we touch, we help and seek help … we congregate. Is it possible to subdue all this activity? Is it possible to limit all of these interactions and still maintain society’s basic functions? This extraordinary question became a reality as political leaders and policy makers confronted the unprecedented COVID-19 pandemic. As they awaited the vaccine programme, social control measures became the primary means of protecting public health. The result, throughout Europe, was an unparalleled programme of public restrictions – lockdowns, curfews, border closures, travel bans, the closure of stores, stadiums and schools, the requirement to work from home, rules on social gatherings and distancing, compulsory mask wearing, hand hygiene, and so on. The paper evaluates the effectiveness of these measures, piecing together evidence from scores of primary sources, using the UK and Spain as case studies. The controls provided limited success, rather than a steady decline in infections, rates fluctuated persistently and rebounded through several waves. The paper seeks to identify the reasons for the erratic progress, the core problem being the sheer complexity of the response. Faced with a monumental life transformation, the citizens of COVID responded in unanticipated and unpredictable ways which, with the benefit of hindsight, it is now possible to see more clearly. The paper concludes with some lessons learned, which may improve the future conduct of crisis management.

## Introduction

We begin by acknowledging that the unprecedented ferocity caused by SARS-CoV-2, the precipitous transmissibility of the virus, and its ability to mutate genetically. Globally, at the time of writing, the pandemic has resulted in 380,321,615 confirmed cases and 5,689,741 deaths ( WHO Coronavirus Dashboard, Feb 2022). All this despite what is often and quite correctly referred to as the largest programme of interventions and the largest expenditure of public resources outside of wartime ( Financial Times, 2020). It is over two years since the onset of the pandemic in December 2019 and throughout the world, inquiries, formal and informal, have begun on the effectiveness of that response ( Talic *et al.*, 2021).

How will history judge the policies and interventions mounted in the attempt to contain the virus? To be sure, there will be much political finger-pointing and partisan name-calling. The scientific response will be calmer and more considered, but it can be said with some confidence that the vaccination programme will be considered the ‘game changer’. Solid, real-world evidence exists on the effectiveness of a range of vaccines in reducing virus transmission, hospitalisation rates and COVID-related deaths (e.g., GOV.UK, 2021d). No policy is perfect, of course, so it is also likely to be acknowledged, both globally and nationally, that the supply and rollout of the vaccines has been remarkably uneven with significant adverse consequence ( Nature, 2021). It is also clear that there has been a considerable drag on progress due to vaccine hesitancy and intense ‘anti-vax’ campaigning ( Benoit & Mauldin, 2021) as well as grossly inequitable vaccine rollout in poorer countries ( Krishtel & Hassan, 2021).

What is far from clear is how history will judge the pandemic management policies mounted, pre-vaccination, in the first 18 months of the worldwide response. These interventions were variously known as ‘lockdown’, ‘mitigations’ or, more prosaically, ‘non-pharmaceutical interventions’ (NPIs) ( Mendez-Brito *et al.*, 2021). Once again, the enormity of this response must be acknowledged. It consisted of a wholesale attempt at behavioural control across entire populations, which demanded huge adaptations to public services across all social domains – not only healthcare but also social care, education, transport, policing, border control, the economy, etc. ( Dodds *et al.*, 2020).

It is striking, looking back at the early days in the spring of 2020, that it was believed that growing levels of COVID-19 infection could be managed by turning on particular packages of NPIs at particular times ( Zhou *et al.*, 2020). Political messaging over this long period was all about the need to add and occasionally to reduce the array of controls in order to maintain control over current rates of infection. We now know that these mitigation strategies struggled to succeed and that non-pharmaceutical aspirations gave way to pharmaceutical actuality. Such a therapeutic paradigm shift, however, leaves behind an intriguing counterfactual question - without the biological discoveries, would the social control strategies, in the long run, have struggled to succeed?

We will never know of course. Thinking about what has not happened but could have happened is the stuff of ‘counterfactual history’ ( Evans, 2014). Despite the fact that such exercises in ‘altered pasts and alternative futures’ are necessarily conjectural, there are important lessons to be learned by posing the question and this is our task in the remainder of the paper. The first section examines European data relating to transmission curves, which reveal how well and how poorly the virus was brought under control. In particular, we contrast the initial expectations and models of how the virus would behave with data on the rolling averages of transmission over the first 18 months of the pandemic. Wave after wave, public restraints and surveillance strategies were implemented, which resulted typically in a start, stop, start, stop pattern of virus control.

The next part of the paper seeks to explain these fluctuation outcomes. Our basic thesis here is that the first-wave policy response was significantly undermined by its own complexity, and we call on the assistance of ‘complexity’ or ‘systems’ theory to support this hypothesis. Much of the development of these ideas has been conducted at high levels of abstraction in methodological journals and it is important to convey that these system dynamics are in fact routine features of all public policy and healthcare programmes. To this end, we produce a simple typology of seven modes of system complexity, showing how each is deeply embedded in the response to COVID-19.

The paper then proceeds by furnishing more detailed illustrations of each of these dynamics in the virus mitigation strategies (NPIs) implemented in the UK and Spain. We restrict our analysis to two countries in the expectation that the mechanisms described will strike a chord with readers familiar with other policy systems across Europe. All manner of controls, guidance, support, and legislation were unleashed, which generated a tangle of contradictory forces, blocked opportunities, displaced effects, and unintended outcomes. Underperformance was the norm. But was this necessary?

This brings us to our conclusion. The world over, the policy track record of tackling ‘wicked problems’ ( Rittel & Weber, 1974) with complex interventions is not impressive. But complexity should be regarded as a challenge rather than an adversary. Our account reveals many failures, but it does suggest opportunities for learning about crisis management and evidence-based policy. These potential gains are described in the final section.

## COVID-19 transmission: expectation and reality

**Figure 1.**
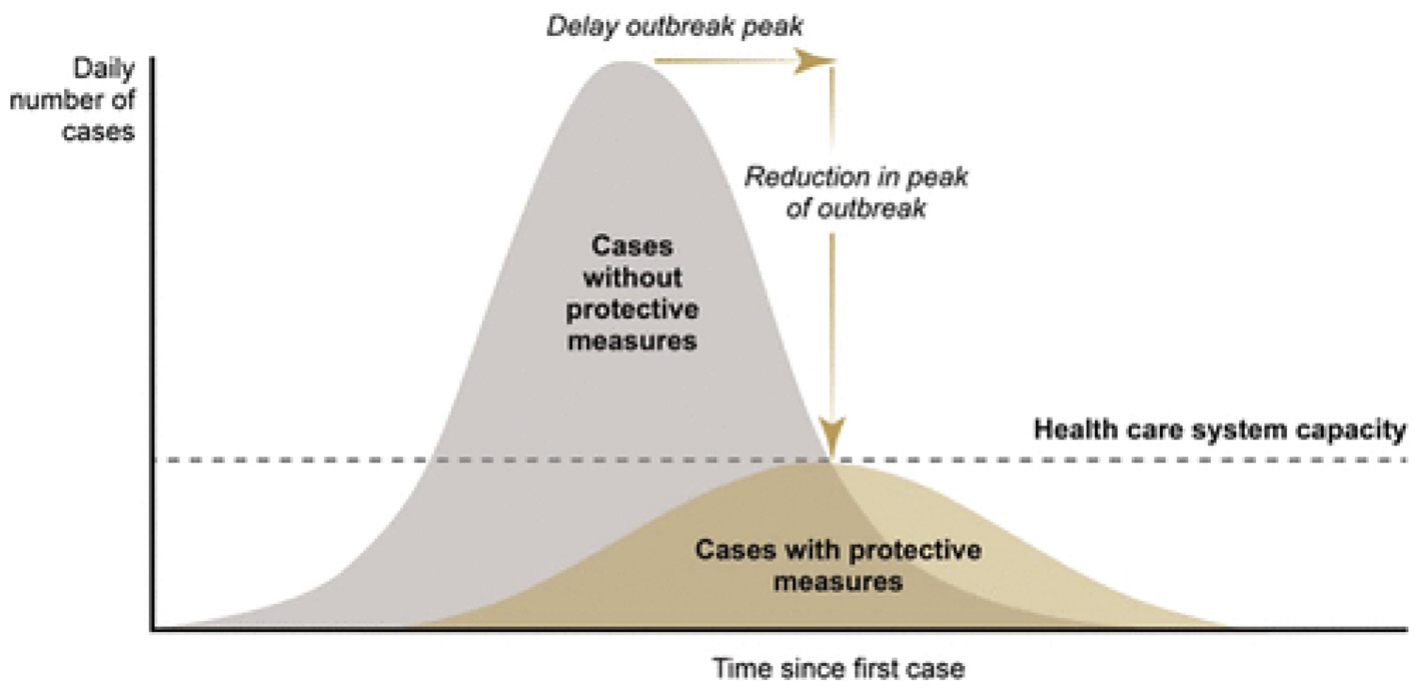
Expectation. Source: Pawson (2021). The coronavirus response: boxed in by models. Evaluation, 27(2), 149-167. https://journals.sagepub.com/doi/full/10.1177/1356389020968579.

In the first stages of the COVID-19 pandemic, political leaders throughout the world were quick to vouchsafe that they would ‘follow the science’ ( Sasse *et al.*, 2020). The science in question, mathematical biology, was hardly known to the public but in the space of a few months the basic concepts and imagery associated with the modelling of infectious disease became remarkably familiar – particularly the phrase capturing the overall aim of ‘flattening the curve’. According to classic epidemiology the basic transmission curve in a major epidemic takes shape as a disease moves through four groups – the susceptible, the infected, the recovered and the deceased. There is a generic pattern, the bell shape, whereby the number of cases increases exponentially until the proportion of the susceptible has been sufficiently depleted (through recovery or death) so that the growth rate then slows and the number of cases drops eventually so that the epidemic is no longer sustained ( Keeling & Danon, 2009). This ‘natural’ shape of transmission graph is illustrated in the upper section of Figure 1, labelled the ‘number of cases without protective measures’ (known colloquially as the ‘do nothing curve’).

The policy response, of course, is to ‘do something’ and this results in the array of interventions aimed at mitigating the spread of the virus – hand hygiene advice, provision of protective equipment, installation of additional ventilators in ICU units, recruitment and training of extra critical care staff, the closure of shops, stadiums and schools, social distancing, curfews, lockdowns, travel bans etc., etc. The challenge facing the mathematical modelers is predict the revised trajectory of the disease under the protective measures using statistical estimates of the collective impact of various permutations of the interventions just mentioned. The expectation was that it is possible to calibrate closely the response that a forward programme of interventions would deliver. Implementation of the optimal set of measures would then flatten the curve, thus reducing disease impact so that casualty rates would not exhaust health system capacity ( Pawson, 2021) (see lower portion of Figure 1).

**Figure 2.**
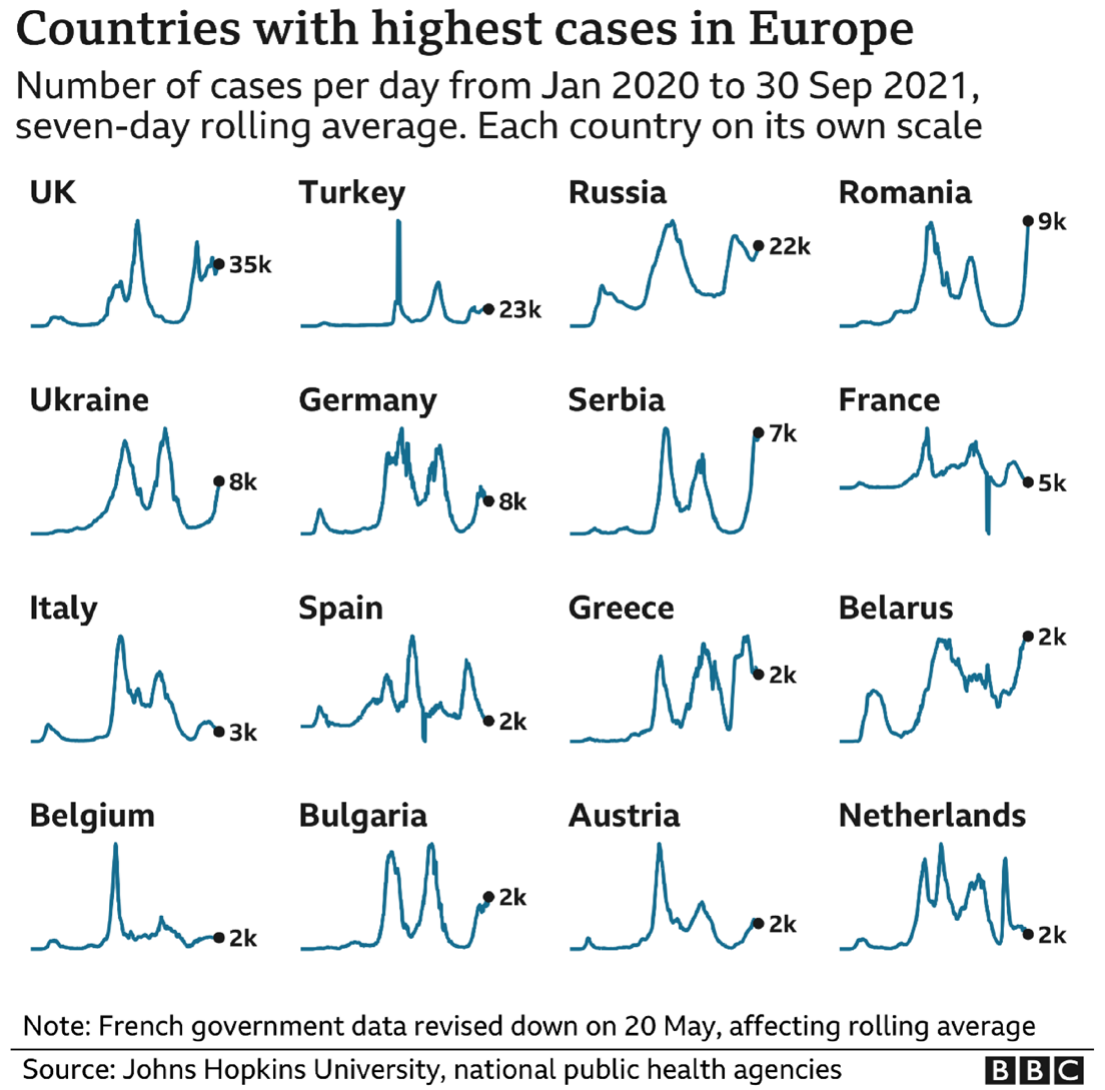
Reality. Source: BBC News https://www.bbc.co.uk/news/uk-58783591.

Policy makers in each European country were in receipt of such advice, the recommended batches of measures were implemented, and the predicted models proved fruitless ( James *et al.*, 2021). The underlying statistical estimates of how the virus could be brought under control were simplistic, based on estimates of how people would respond to each potential measures, rather than treating that response as an adapting, self-transforming whole system ( Pawson, 2021). Much of the real reaction, negotiation, fatigue, dispute, confusion and sheer muddle produced by the NPIs is thus lost on the models. We provide evidence for these bold claims in a subsequent section but first it necessary to explore the real shape of COVID-19 transmission. Figure 2 with its mountainous landscapes of infections provides a stark contrast with the aspirations in Figure 1.

This is one of the renowned John Hopkins ‘dashboard maps’ (2021), relating specifically to the pre-vaccination period. It is important to note that the graphs in Figure 2 are not to scale and that only incidence is mapped. Death and hospitalisation rates also fluctuate just as significantly but with a different rhythm. Data collection methodology and operationalisation (and rigour) also vary from country to country. Two common features stare out from the rolling averages and provide the focus for the reminder of the paper. 1) rather than a steady rise and decline in infections, rates fluctuated persistently and rebounded through several waves. 2) management of the virus was never fully achieved through social controls but only began to improve with the arrival of the vaccination programme in late 2021. How can we begin to explain these uncontrolled fluxes?

## Applying complexity theory to the COVID-19 response

The thesis here is that NPI policy regimes across Europe were undermined by their own complexity. There has been a significant ‘turn’ towards complexity and systems thinking across the social sciences in recent years ( Williams, 2021) and this orientation features increasingly in policy analysis ( Daviter, 2019), in implementation science ( Braithwaite *et al.*, 2018), and most significantly in healthcare, via the UK Medicinal Research Council’s guidance on the evaluation of complex interventions ( Skivington *et al.*, 2021). Complex systems are self-transformative, driven by an ecology of agents who interact, compete, and adapt. Novel outcomes emerge constantly, generating all manner of unintended consequences. The properties of complex systems are dissected in great detail in the MRC framework (adaptation, emergence, perverse consequences, feedback loops, blockage points and structures, non-linearity, contextuality, tipping points, path dependency, openness, self-transformation, etc.). We simplify this analysis here, identifying seven key modes of system complexity ( Table 1). For each dynamic, we provide a brief discussion of how it was embedded within pandemic crisis management.

**Table 1.** Modes of complexity.

*Mode 1: Disparate * *command and * *control systems*	This mode of complexity occurs in social systems managed under multiple ‘hubs’ and ‘relays’. Most national responses to the COVID-19 pandemic operate with a measure of regional and administrative independence and with long implementation chains. Rather than a uniform unfolding of policy a ‘system of policy systems’ develops. Complexity multiplies according to the connectedness of the networks of control. The more autonomous the network of hubs and relays, the more likely that inconsistencies, contradictions, and conflicts will ensue.
*Mode 2: * *Interaction and * *emergence*	Complex policy systems utilise many different measures. In the case of COVID-19 the controls applied include hand hygiene, protective equipment, closing of stores, stadiums and schools, rules on social distancing, curfews, travel restrictions, working from home, etc. The effects of these actions are not additive but interactive. Each intervention conditions the others, often in unanticipated ways. The combination of measures generates emergent effects, which may complement each other but also can reduce, compete with, or displace the intended effect.
*Mode 3: Policy * *discord and moral * *disharmony*	It is impossible to separate complex interventions from the human agency required for their delivery. Draconian restrictions on everyday activity meet with diverse responses from different sections of the public, with the potential to create a moral struggle between those who ‘care’ about complying with controls and those who ‘do not care’. The wayward actions of notable ‘free riders’ , who flout the restrictions may cause a tipping point leading to a sharp and unpredictable increase in moral disharmony.
*Mode 4: * *Contextual * *heterogeneity*	Complex interventions always create unequal outcomes. The COVID-19 pandemic does not affect everyone uniformly – with vulnerable groups, such as ethnic minorities, the elderly, those with chronic diseases and disabilities, those living off the informal economy, etc. being hit the hardest. Some of the measures designed to reduce infection and death, by ignoring such contextual heterogeneity, had the perverse effect of intensifying health inequalities and reducing the impact of controls.
*Mode 5: Ambiguity * *in regulations and * *guidelines*	Policies begin life as text in the form of regulations or guidelines. In complex, hastily assembled interventions like COVID-19, some ambiguity in official documentation is inevitable, with unclear pronouncements introducing further diversity in the public response. Uncertainty arises because of: i) the legal status of the guidance - what is law and what is merely advisory? ii) ambiguities in the wording or phrasing of the guidance, iii) the remoteness of official communication channels from everyday behaviour.
*Mode 6: Temporal* *change in public * *attitudes*	Public allegiance and adherence to complex interventions vary over time. There is a typical rhythm to public commitment – often moving from enthusiasm, to acceptance, to routinisation, to fatigue. This progression is pronounced, as in the COVID-19 restrictions, when unprecedently severe controls are maintained over a considerable period of time. The pace of change in such motivational patterns is not predictable and not under the control of policy makers.
*Mode 7: Exit and * *sustainability * *effects*	Sustainability represents one of the greatest challenges in healthcare policy – what happens when interventions wind down? In the case of COVID-19, the expectation was that social controls may be removed once infection and death rates have diminished sufficiently. The easing of restrictions is itself a significant policy manoeuvre and carries all of the uncertainties and complexities associated with their introduction. The timing and execution of exit strategies is thus unpredictable; the relaxation of measures often generate ‘rebound effects’.

## Complexity in pandemic management in the UK and Spain

The paper proceeds by furnishing some detailed illustrations of each of these dynamics in the virus mitigation strategies (NPIs) as implemented in the UK and Spain. The methodology used here is based on ‘realist review’ a method of research syntheses which collects together evidence on programme theories or explanatory themes ( Pawson, 2006). Here we present a 'rapid review', highlighting just a few key instances of each mode of complexity ( Saul *et al.*, 2013). For each of the modes of complexity shown in Table 1, examples from the United Kingdom and Spain are uncovered, with the aim of promoting debate. The process uncovered in our analysis go a long way to explaining the halting and intermittent nature of virus control as described in Figure 2. Although the analysis is restricted to two countries, the mechanisms described will strike a chord with readers familiar with other policy systems across Europe.

## A Methodological Note on Rapid Review

Realist synthesis (RS) was developed at book length by Pawson (2006). A number of extensions and clarifications followed, most notably a formal account of publication standards by Wong *et al*. (2013) and a practical guide for information specialists ( Booth *et al.,* 2018). RS is a method specially designed to review the evidence on complex social interventions. Accordingly, it has found much use in public health research, typified by its usage here in evaluating the effectiveness of the unprecedent magnitude, expenditure, duration, and complexity of the measures designed to prevent the spread of the coronavirus infection.

The methodology of evidence synthesis has blossomed in recent decades. The founding approach, known as ‘systematic review’ or ‘meta-analysis’, originated in medical research, and sought to provide a statistical estimate of the mean effect of a particular treatment or therapy across many different clinical trials. The effectiveness of social interventions is much more difficult to capture in this singular fashion - because they vary significantly in their design, management, and implementation; because they vary markedly in their effectiveness in different communities, sub-populations, cultures and time-periods; and because they are evaluated in primary studies that utilise decidedly different research methods. The ‘mean effect’ is meaningless under such a scenario. Accordingly, many new methods of evidence review have proliferated ( Sutton *et al*., 2019), one of which is realist synthesis.

The purpose of RS is explanatory, it seeks to understand for whom, in what contexts, and in what respects an intervention works and above all why. It assumes there will be successes and failures and seeks to ascertain why. The review sequence can be summarised as follows: 1. Search and ascertain material on the programme theories, the ideas underlying the intention. 2. Seek primary studies that provide a test of those theories and extract the pertinent data. These primary sources may utilise any and all social research approaches. 3. Assess the rigor and relevance of each piece primary research. 4. Synthesise the emerging findings to assess in what respects the programme expectations are met and in what respects they may fall short. 5. Disseminate the findings, with an assessment of their generalisability and policy implications, as well as a statement on the limitations of the synthesis.

These steps are followed in the above account, though it must be emphasized that this is a ‘rapid review’. It is rapid in several senses – I) The primary literature is gargantuan and so beyond any single summary; one 2021 estimate traces more than 87,000 scientific papers in many different languages on the coronavirus pandemic ( Grabmeier, 2021). II) Pawson and Manzano’s contribution to the study were unfunded and the review was relatively under-resourced. III. It was completed in something like real time rather than, as is more typical, making retrospective comparisons across the history of a family of interventions. IV) At paper length, the reported findings reduce and condense the extracted primary materials, covering only a handful of the most compelling illustrations. V) As with all rapid reviews, the conclusions are thus suggestive rather than in any sense definitive. Indeed, they anticipate the possibility of rival interpretations and the primary goal is ‘to open the debate’.

We believe our review is ground-breaking in its use of complexity theory as the lens through which to understand the mixed fortunes of an intervention. Normally review step 1 uses material extracted from the accounts of programme architects and policy makers. The usage of complexity theory is justified in the main text (basically because the response to the pandemic touched every corner of society). But note, as a consequence, that this RS involved a preliminary step of reviewing the complexity literature itself to ascertain which of its many accounts and dimensions would feed into and best support a public health study.

The overall research design was devised by Pawson. He selected and reviewed the material used in the UK section of the study. Serrano Gallardo and Manzano conducted the parallel review on the Spanish response, calling on and inserting further materials from their own experience of evaluating public health programmes.

## Mode 1: disparate command and control systems

Spain is a country administratively divided into 17 autonomous communities and two autonomous cities, which have considerable governmental independence, both at the legislative, financial and executive levels. The Spanish Constitution grants the government the power of declaring a ‘state of alarm’ in exceptional emergency circumstances, giving the national government authority to overcome the devolved powers. At the time of writing, it had been used three times during the pandemic: first strict confinement (March to June 2020); confinement of the autonomous community of Madrid (October 2020); and national night curfew (from 11pm-6am) from October 2020 to May 2021.

The bigger picture, however, is that guidelines and mandatory regulations have been issued by the central state and by the regional governments of the 17 autonomies. Accordingly, some autonomous communities followed the actions recommended by government but not others and, significantly, the recommendations were implemented at different times (early vs late adopters). For example, Madrid did not close bars and restaurants at all following the conclusion of the stringent national home confinement in June 2020, despite showing infections and mortality rates well above of the national average ( Instituto de Salud Carlos III. Centro Nacional de Epidemiología, 2021). By contrast, Extremadura (western region bordering Portugal) maintained the longest bar and restaurant closure, achieving the lowest COVID-19 incidence rate in Spain after the third wave ( Manchado, 2021).

**Figure 3.**
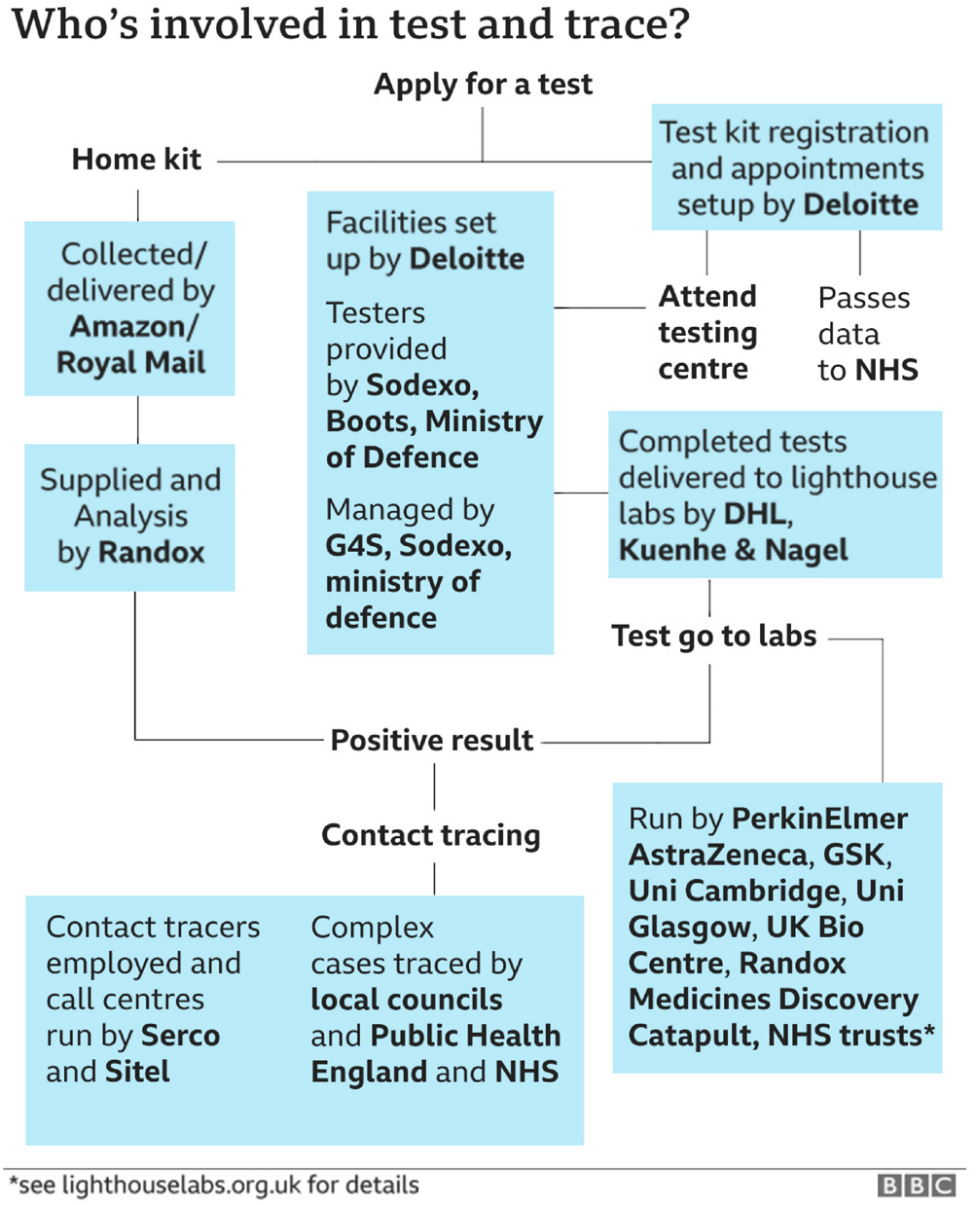
Cooperate responsibilities for UK COVID tracking. Source: BBC News https://www.bbc.co.uk/news/health-55008133.

In the UK, health policy is also a devolved matter, with England, Scotland, Wales and Northern Ireland having their own mandates. In this instance there is little difference between the chosen package of measures; the only variations being modest disagreements on their optimal timing ( Institute for Government, 2021a). However, the main frictions in control powers in the UK are to be found within specific delivery systems. Figure 3 pictures how the UK Department of Health and Social Care (DHSC) funded but then ceded management responsibility for its ‘Test and Trace’ system to the private sector ( Triggle *et al.*, 2020).

As can be seen more than two dozen agencies are involved, mostly private companies, each with its own priorities and agendas. It provides a classic example of a system with an over-complex command and control structure. Subsequently, the National Audit Office (2020) reported on a catalogue of complications and conflicts. For instance, the freshy appointed contact tracers (to a large extent government funded) were vastly underutilised (4% of contracted time). Targets were missed routinely, the DHSC had assumed that each case transferred to the tracing system would provide 10 to 30 contacts (the actual number was 2.4). Detailed analysis of the Spanish contact tracing system reveals a similarly labyrinthine implementation map with similarly disappointing detection rates ( De Vega, 2020) ( Alonso & Larraz, 2021). Too many cooks spoiled the broth – substantial public resources were wasted.

## Mode 2: interaction and emergence

Estimates of the impact of individual prevention measures often proved erroneous, sometimes with deadly effect. All interventions, however well intentioned, depend for their impact on what happens elsewhere in the system. In the first wave of the UK virus, the high risk of hospital acquired infection ( Heneghan *et al.*, 2020) and the urgent need for more hospital space to treat COVID patients led to a programme of discharging elderly patients to care homes. This strategy succeeded in its primary, numerical aim but failed to include a testing programme to accompany transfer and so displaced the problem causing a substantial surge in care home transmission. Thirty-three care home outbreaks in the first week of March 2020 turned into 793 by the end of the month ( GOV.UK, 2020). Increasing COVID-19 space and services in hospitals also led to substantial shortfalls in routine and planned care. Cancer services were significantly affected with a growing backlog for referrals as well as delays and cancellations in first treatments ( Macmillan Cancer Support, 2020).

In Spain, the massive spend on COVID-19 care also syphoned resources from other parts of the healthcare system – but with some subtle differences. Many primary healthcare centres were closed during the first wave, many have reopened since, but in many of these centres, services remained rationed and suboptimal. Once again, other services such as home healthcare were substantially reduced and waiting lists for outpatients and elective surgery increased. During the first wave, the vast majority of nursing home residents who fell ill with COVID-19 in Spain were not transferred to hospitals, but remained in the same nursing homes, which had neither the resources, nor the knowledge, nor the abilities to properly handle the situation. The highest percentage (66%) of deaths from COVID-19 occurred in nursing homes ( Costa-Font *et al.*, 2021). Once again, this phenomenon was not the same throughout the national territory. Excess mortality was registered in 7 of the 17 autonomous communities, which ranged from 10.3% in Castilla-León to 52.5% in Madrid. On the contrary, in 10 autonomous communities there was a decrease in the mortality of elderly people in nursing homes, from 3.8% in Extremadura to 86% in the Canary Islands ( Zunzunegui, 2021).

Across both of our miniature case studies, the implementation of protective measures involves a transfer of resources from other services, with unforeseen and damaging consequence elsewhere in the healthcare system. But this is not a given. It is evident in some rare instances in Spain that opportunities have been grasped for damage limitation. 

## Mode 3: policy discord and moral disharmony

The public mood and support for government COVID-19 policy follows a similar trajectory in the two nations. ‘Free riders’ are people who gain from the collective effort, without bothering to make an individual contribution. The activities of prominent free riders had a deleterious effect on UK public trust in the management of the epidemic. A longitudinal survey by Fancourt *et al.* (2020) charts the changes in public trust in the government handling of the pandemic. Starting in May 2020 there was a steep decrease in confidence, which never recovered (until the vaccination programme). This date coincides with the discovery that Dominic Cummings, the Prime Minister’s then closest advisor, had broken lockdown rules with a 500-mile round trip to a family estate. In Spain, the most notorious case involved an entire group of free riders attending a major gala dinner in an enclosed space. Multiple political worthies gathered including the health minister, masks were not worn, and social distancing rules were ignored ( Valdeolivas, 2020).

The fact that such high-profile officials had abstained from collective responsibility ignited a torrent of media abuse in the two countries – ‘one rule for those in charge and one rule for everyone else’. After these high-profile incidents, the negative and lasting decline in public confidence was further exacerbated by crowds of anonymous free riders who gathered in all manner of communal spaces. Trust in the government was lost progressively. According to the Spanish Sociological Research Centre in April 2020, 73% of the population believed that the government was able to manage the pandemic ( Centro de Investigaciones Sociológicas, 2020), whilst in February 2021 only 18.5% held similar views ( Centro de Investigaciones Sociológicas, 2021). Longitudinal polling by Ipsos MORI details a similar substantial decline in the same period in support for the UK government’s handling of the outbreak ( Health Foundation, 2020).

The blame game continued in both countries. In Spain, typically demonised groups such as the youth ( Lledó, 2020) or migrant populations ( Radiotelevisión española, 2020a) were accused of driving up infections and deaths. In the UK, the growing complacency of young people incited both media condemnation (‘the covidiots’) and empathy (‘don’t scapegoat the young’) but, once again, the implications are crucial ( Reicher & Drury, 2021).

The explanation for rising resistance to official advice rests in a phenomenon called ‘risk normalisation’ – small risks become increasingly acceptable over time ( Murphy, 2020). Despite year-long warnings of the savage consequences of COVID-19, most young people had no direct experience of the misery it could cause, many will have noted the limited and sporadic deterrence offered by police, and a few of them may have come across the official reports on the minute death and serious illness rates in their cohort ( Bhopal *et al.*, 2021). Putting such factors together anticipates and provides further explanation of the continuing struggle to sustain lockdown policies.

## Mode 4: contextual heterogeneity

National policies often fail to get to grips with local complexity and this is demonstrated in the limited reach of the NPIs in the UK in respect of black and minority ethnic (BAME) communities. Prevalence, mortality, and shielding rates can be pinpointed minutely at the ‘ward’ level and these show the persistent toll of the virus on areas with high proportions of BAME residents ( Otu *et al.*, 2020). Local, ‘soft intelligence’ identifies why these communities fared badly – e.g., collapse of the local ‘cash-in-hand’ economy, significant exposure to ‘fake news’ media, cultural misunderstandings with providers and referral systems, reliance on public transport, social distancing problems with large families in small houses, curtailment of funeral and mourning services, and so on ( West Yorkshire and Harrogate Health and Care Partnership, 2020). Broad-brush, top-down national programming can never counter such deep and locally rooted mores.

It is important to note that Spain already had a high incidence of unemployment even before the 2008 crisis. Inequality in gross income per capita is also high ( Anghel *et al.*, 2018) and the informal economy, estimated at 17.2% of GDP in 2017, is still considered very substantial ( Deléchat & Medina, 2021). This context was nothing but conducive to further exacerbating inequalities in COVID-19. The Spanish national COVID-19 survey revealed how the most affected sectors of the population were those with lower incomes, migrant women and those who worked in the informal economy and in more precarious sectors such as cleaning, home care and nursing homes ( Carlos III Health Institute, 2020). During the first wave (March-May 2020), Barcelona neighbourhoods with lower incomes had a 42% higher incidence of infection than those with higher incomes ( Amengual-Moreno *et al.*, 2020). The crucial point here is that well intentioned national COVID-19 policies do not just reproduce but actually intensify existing inequalities. The poorest communities are more affected by job losses as the economy shrinks ( World Health Organization, 2021). Allas *et al.* (2020) made an early estimate of UK unemployment risk as follows: ‘The proportion of jobs at risk in ‘elementary occupations’—which employed 3.3 million people in 2019 and include jobs such as cleaners, kitchen assistants, waiters, and bar staff—is around 44 percent. In contrast, the same number for professional occupations—such as computer programmers, project managers, and accountants—is around five percent’. What is more, the UK jobs that remain lower paid (health and social care, transport, shops) are mainly ‘public facing’ and thus carry elevated risks from the virus ( Office for National Statistics, 2020).

Spain has a welfare model that relies heavily on family responsibilities, and different generations co-habit together for longer than anywhere else in Europe. The ‘kinship solidarity model’ characteristic of Southern Europe countries is based on an asymmetrical gender division of work where women’s care is essential for the provision of welfare ( Minguez, 2017). Strict lockdown measures resulted in many of these informal support networks being reduced or suspended, undermining still further the protection afforded to poorer communities ( Amnistía Internacional, 2021).

## Mode 5: ambiguity in regulations and guidelines

COVID-19 policy imposes a mass of restrictions on normal behaviour. These restrictions are delivered in the form of official communications on which activities are permitted and which are restricted. Ambiguity and inconsistency in these guidelines are inevitable, with unclear pronouncements introducing further diversity in the public response. Government announcements in both counties blurred the distinction between ‘law’ and ‘guidance’ and ‘exhortation’ in the coronavirus regulations, creating potential confusion among the public. In the UK, the key message in the original government documentation on lockdown read as follows: ‘ *What you can and cannot do during the national lockdown. You must stay at home. The single most important action we can take is to stay at home to protect the NHS and save lives. You should follow this guidance immediately. This is the law’* Hickman (2021). The author goes on to point out that much of what is stated in the so-called law is actually public health advice. Many exceptions to the ‘law’ were in fact permitted - shopping for essentials; allowances for childcare bubbles; working where it is "unreasonable" to work from home; medical appointments and emergencies; moving to a new house; daily exercise, etc.

Some of the key concepts in government edicts remained inscrutable. One key task was to restrict social interactions but without forcing individuals and families into isolation. In both countries this task was made manifest in the idea that association was only allowed in ‘bubbles’. Bubbles proved as imperceptible as hot air – with whom, how, and where might they form? Schott (2020) provides a detailed study of ‘graphic confusions’ in UK Government’s COVID-19 official communications. One baffling example concerns a poster explaining the rules on meetings in which the public is permitted a choice: ‘Your household can meet up with *one other household* indoors or outdoors’ OR ‘You can meet up in a group of up to *six people*, outdoors only’. The terminological turmoil in this respect is nothing compared to the erratic Spanish rules over the Christmas period 2020 when each of the 17 autonomous community and the two autonomous cities proposed a different welcome. Regulations differed on whether ‘relatives’ or ‘close friends’ were allowed to gather. The recommended boundaries ranged from 6 in Galicia in a ‘single specified bubble’ to 10 in Ceuta in ‘any number of bubbles’ ( Radiotelevisión española, 2020b).

Once again, we glimpse an unintended consequence, inconsistency across different recommendations became a barrier to compliance. Initially in Spain there was much public consternation about the mandatory use of masks outdoors, especially in children’s playgrounds, when there was no such requirement in enclosed closed places such as bars and restaurants ( Royo-Bordonada *et al.*, 2021). The inconsistency of messaging becomes even more problematic when restrictions are turned on and off, and then on and off again. Analysis by Dixon & Roberts (2020) showed that there were two hundred rule changes by as early as September 2020 in the UK. Whilst such tinkering was perfectly understandable it was reflected in measurable levels of public confusion ( Fancourt *et al.*, 2020).

In the last analysis, the sheer impracticality of narrative control over micro social interaction is reflected in the use of some familiar slogans. COVID-19 restrictions extended to most walks of life and exemptions were always included. They are often so potentially compendious, however, that they have to be captured in stock caveats such ‘essential activities’, ‘reasonable excuses’, ‘where necessary’ or ‘force majeure’. Knowing exactly where to ‘draw the line’ thus becomes problematic for officials and the public. As a consequence, public pronouncements also swung gradually to emphasising the importance of ‘behaving in responsible manner’, ‘taking great care’, ‘staying alert’ and so on. Risk management was shifted subtly and arbitrarily onto the individual, with no foreknowledge of whether this new emphasis could be effective ( McVeigh & MacLachlan, 2021).

## Mode 6: temporal change in public attitudes

Social interventions often generate an initial surge of enthusiasm with the introduction of innovative ideas (the novelty effect) ( Bracht & Glass, 1968). There is also some pride involved in being in at the beginning of a significant initiative (the showcasing effect) ( Chiesa & Hobbs, 2008). These sensations often dissipate over time as programme activities fade into the background (the routinisation effect) ( Karo & Kattel, 2018). As time continues, policy expectations may become tiresome or even resented (the fatigue effect) ( World Health Organization, 2020). Such a pattern is discernible in the response to COVID-19 restrictions in both countries.

In the UK, the remarkable ‘Clap for Carers’ event in which neighbours stood on their doorsteps clapping their hands and banging pots and pans every Thursday at 8pm, represented a significant, if ‘un-British’, show of public affection for health workers battling against the virus ( Manthorpe *et al.*, 2022). Spaniards were even more spontaneous, clapping in their balconies every day at 8pm ( Cabedo-Mas *et al.*, 2021). But slowly stamina declined, and streets and balconies became empty.

In terms of the medium and long term; there is some evidence of the routinisation effect, when people seek to push back rather than withdraw under restrictions. This process is demonstrated in the significant differences in the numbers of children claiming exemptions in order to attend schools in the UK in the two periods of formal ‘closure’. The Department for Education ( GOV.UK, 2021a) reported that 21% of primary school pupils and 5% of secondary school pupils went into school in January 2021. This compares with 4% of state primary school pupils and 1% from state secondaries who were in school during the closure in the previous year.

A stiffer measure of resistance then follows in both countries. In Spain, according to a March 2021 ISCIII COSMO survey, reported by Instituto de Salud Carlos III (2021) there was a decrease in the frequency of compliance with all preventative measures as pandemic fatigue increased. This decline was particularly discernible in the answers to motivational questions such as ‘I am losing the will to fight against COVID-19’, ‘I am tired of the debates about COVID-19’ and ‘I am tired of hearing about COVID-19’.

Polling by the Scottish Government (2021) showed that in October and November 2020, 4 out of 10 parents of under-18s admitted to adapting COVID-19 guidance to suit their family needs. One example was that 19% agreed that ‘It’s okay for my child(ren) to go into their friend’s house if I don’t go in with them’. The main reasons provided by parents for adapting the guidance were; the mental health of their children (41%), followed by applying common sense (35%), to help improve their own mental health (30%) and to allow them to work (26%).

## Mode 7: exit and sustainability effects

It is relatively easy to invent policies that restrict social interaction. But not quite so easy to implement and evaluate them, as we have seen to this point. Uncertainty actually increases when it comes to deciding how and when to withdraw them.

Closing schools, shops, theatres and so on was much simpler to implement than reopening them with capacity limitations, one-way systems, sanitising points, screening and booking systems. In lifting the first lockdown, UK Government advice ( GOV.UK, 2021c) for retailers included: completing a COVID-19 risk assessment, cleaning more often, reminding customers and staff to wear face coverings, ensuring social distancing, improving ventilation, taking part in Test and Trace, turning away people with coronavirus symptoms and attending to staff mental health needs. Additionally, some establishments were expected to keep records of all visitors, to reduce capacity, to manage queues, to erect barriers and screens to protect staff. Identical requirements were placed on many establishments in Spain ( Consejo Interterritorial. Sistema Nacional de Salud, 2021).

These are arduous expectations, especially for small businesses. Very little evidence is available on how well these requirements were met. Data is still gathering on one important consequence – how many small businesses will survive the roundabout of restriction and derestriction? How many temporary small business closures will become permanent? Estimates of UK impact can be found in a Registry Trust report (2021). Big business has proved more reliant under the pandemic but even here there are casualties, one of which impacts on our case comparisons. Nissan made the decision to close its plant in Barcelona with a loss of 2,800 jobs but removed a short-term threat to the 6,700 at its Sunderland plant in the UK ( Euronews, 2020).

These evidential glimmers lead us to our final consideration on complexity. We have argued that the effectiveness of any particular measure depends on its interaction with all other measures (Mode 2). When it comes to lifting COVID-19 restrictions, political and economic interests fight against heath considerations as never before. As a summary statement, this conclusion from the UK Institute for Government report (2021b): serves well:

‘ *When and how to start lifting lockdown will present the prime minister and his cabinet with some of the toughest choices they will ever have to make … At the start of the crisis, what was good for public health was also probably in the economy’s long-term interests. As we move into the next phase there is a balance ministers will need to manage – they will be walking a tightrope between the risks of another surge of infections and lasting harm to the economy, people’s lives, livelihoods and prospects*.’

## Conclusion

In the introduction we posed the question - without the biological discoveries, would the social control strategies, in the long run, have struggled to succeed? We warned that it was impossible to settle such counterfactual questions, that piecing together evidence would be hazardous, but that informed conjecture on the effectiveness of NPIs would be useful in learning about their application in future crisis management.

It is not only the public that suffers pandemic fatigue but so do commentators and policymakers. Much of the preliminary evidence that we have pieced together is in danger of being forgotten. Before the advent of the vaccination programmes, very few European governments felt able to boast of the sustained reduction of the virus and the reasons for this reluctance should not be lost to history ( Goniewicz *et al.*, 2020). Many years ago, Lindblom (1959) characterised policy making as ‘muddling through’ and, alas, this description applies all too well to the efforts to contain the virus.

The boundaries of our analysis should be made clear. Long-term lockdown (extended NPIs) may well work more effectively in countries with authoritarian governments, compliant populations, and mass surveillance systems – though accessing uncensored evidence is difficult ( Thomson & Ip, 2020). New Zealand’s famed exceptionalism, pursuing a zero COVID-19 strategy and implementing an initial stringent and brief lockdown ( Robert, 2020) also has distinctive roots: geographic isolation, easy and immediate border closure, a unitary system of government and a tiny population - the so-called ‘team of five million’ ( Baker *et al.*, 2020). But in complex, liberal democracies, with diverse and disputatious populations, with instant and endless social interaction, with extensive worldwide interconnectedness, and with dispersed administrative systems, the broad picture is that centralised control systems generated outcomes that were partial, short-lived, and indeterminate. Modern European social life is perfectly organised in ways that multiply the microcircuits of disease transmission. 

We should not have been surprised. State public policy, as McConnell and Stark (2021) advise, is often imprecise and fractured. Accordingly, the same two-steps-forward, one step-backwards pattern of progress is familiar right across the policy waterfront. Most famously, economic policy over a century has utterly failed to prevent cycles of ‘boom and bust’ ( Quinn & Turner, 2020). ‘Crime waves’ reoccur as prevention interventions falter with offenders adapting deftly to successive countermeasures, whilst fresh opportunities continue to arise ( Sacco, 2005). Studies of comparative, long-term trends in social mobility reveal little more than trendless fluctuation, the so-called ‘constant flux’ pattern ( Erickson & Goldthorpe, 1992). Many foreign aid projects are turned off on because of the ‘Samaritan’s dilemma’, with some recipients choosing it to use the support to improve their situation with others relying on it as a means of survival ( Buchanan, 1975). The very many schemes devised for managing demand for healthcare tend to meet with immediate success only to be met by ‘rebound effects’ due to patients’ steadily rising expectations and the constant advances in treatment technology ( King’s Fund, 2016). In all of these examples the intended effects dissipate and fluctuate as they are met with counteracting mechanisms akin to those analysed above. Complex systems are perfectly designed to achieve the outcomes that emerge.

We arrive at our counterfactual conclusion. Across Europe, in the absence of the vaccination programmes, NPIs would have failed to quell successive virus waves. Countless excess deaths would have resulted until infection rates became sufficient to reach the threshold of herd immunity. One cannot, of course, provide direct evidence for something that did not happen but there is a glimpse of this alternative future in UK data on infection rates that stretch into the vaccination period. Daily case rates in October 2021 reached 44,890, a figure broadly comparable with transmission at the pre-vaccination summit ( GOV.UK, 2021b). This suggests that all the social dynamics of COVID-19 transmission remained active and uncontrolled, the all-important difference being that, thanks to vaccination, death and hospitalisation rates diminished significantly from their previous peaks. And, thankfully, they continue to do so.

Despite this pessimistic conclusion, throughout Europe, official inquiries are underway seeking to learn lessons for the future of pandemic management (e.g., UK Parliament, 2021). We hope that some insights from complexity theory find their way into such post-mortems, for its insights are not automatically negative. Complexity should be regarded as a challenge rather than an adversary. To this end, we conclude with some modest recommendations of our own to improve the design and application of NPIs in epidemics and pandemics.

The first is to widen the pool of evidence considered in the manufacture of policy. Consider the status of the material collected here on the seven confounding mechanisms embedded in complex policy systems. It is clearly *not* the evidence that found its way into policymaking. Yet it is perfectly valid and highly reproducible evidence – the problem being that it is often dismissed as ‘critique’. We have called on all manner evidence produced from opinion surveys, from administrative records, from document analysis, from local soft intelligence, from media research, and from accounting and audit. This first call mirrors other significant pleas to construct a wider platform for evidence-based policy – c.f. Sridharan and Nakaima (2012) on the need for an ‘ecology of evidence’ and the UK Medical Research Council’s manifesto for ‘methodological pluralism’ ( Skivington *et al.*, 2021).

The second petition is a corollary of the first. The membership of advisory bodies responsible for producing, interpreting, and advising on current evidence should also reflect the substance of the policies and interventions under development. NPIs, as their name suggests, consists entirely of social interventions and organisational reforms – and yet clinicians, modelers and epidemiologists rather than programme evaluators, implementation scientists, policy analysts dominated the ranks of senior advisors in the UK Scientific Advisory Group on Emergencies ( Thacker, 2020). By contrast, and although Spain has a Coordination Centre for Health Alerts and Emergencies and the Interterritorial Council of the National Health System that advises on public health, the Spanish Parliament (Cortes Generales) does not have any permanent legislative scientific and technological advice mechanism to inform debate and the policymaking process ( Aiello *et al.*, 2020). We counsel on a happy medium. Broadly based expertise is the cornerstone of evidence-based policy.

A third recommendation is to ponder alternative models for the conduct of expert committees – minority reports, tribunal systems, open deliberations, adversarial courts, citizens’ assemblies and so on ^1^. Practical details vary but the underlying principle is paramount: ‘Creating institutions that establish norms and expectations of legitimate disagreement as part of the process of forming and communicating expert advice would make it easier for experts to stay true to their expertise and harder for politicians to hide their judgments behind the science’.

A fourth suggestion is for increased delegation. European COVID-19 policy making has largely preferred evidence with a macro focus, eying the dashboards for telling perturbations. This inclination generated the stuttering stream of national restrictions, described above, based on overzealous extrapolations of shifts in *aggregate data*. There should be more emphasis on the micro-circuits of transmission ( Manzo, 2020), seeking and targeting continuous quality improvement within the many sub-processes, logistics and agencies that embody the everyday response to the virus. Total control of a complex system is impossible but there was considerable local learning on infection control in hospitals, care homes, local communities, policing etc. NPIs sought to modify everyday behaviour. Narrative and exemplars can be better than rules and regulations at inspiring change in commonplace routines ( Greenhalgh, 2020).

A final important plea, is for improved institutional memory. It is true but somewhat oversimplistic to assume, that the vital lessons about COVID-19 policy could have been assimilated from knowledge of previous epidemics ( Tsuei, 2020). But there is at least a case for saying that mistakes can always be avoided by not repeating them. We pointed earlier in the paper to the damage that prominent ‘free riders’ can inflict on public confidence in virus control leadership. Astonishingly, in the UK case, this lesson was immediately forgotten as the aforementioned ‘Cummings effect’ was then followed by the ‘Partygate’ scandal ( Foreign Policy, 2022). A similar affront can be observed in the Spanish context. In the same week that the seventh report of the Rafael Campalans Foundation was released, reflecting the disastrous management of the pandemic, especially in nursing homes ( Precedo, 2022), the scandal of the commissions received by the brother of the President of the Community of Madrid has reinforced public displeasure ( Rubio Hancock, 2022). In the last analysis, pandemic policy is in the hands of political leaders and unforeseen consequences always lurk. Lindblom (1959) was correct in assuming that policy making in complex environments always involves ‘muddling through’. But if we read him carefully and focus attention on bespoke evidence on sub-process and local conditions, he was also correct to insist that there can also be a ‘science of muddling through’.

## Data availability

All data underlying the results are available as part of the article and no additional source data are required.

## Ethics and consent

Ethical approval and consent were not required.
