# Supplementary material for: Non-pharmaceutical interventions during COVID-19 in the UK and Spain: a rapid realist review
Source: Open Res Eur. 2022 Aug 26;2:52. Originally published 2022 Apr 29. [Version 2] doi: 10.12688/openreseurope.14566.2 (PMC10446037; doi:10.12688/openreseurope.14566.2)
Supplement: Peer review history

# Peer review: Reviewer response for version 2

**DOI:** 10.21956/openreseurope.16244.r29984

**Published:** 2022-09-05

**Authors:** Axel Kaehne

**Affiliations:** 1 Health Services Research, Edge Hill University, Lancashire, UK

Many thanks to the authors for their thoughtful response to my comments and suggestions on their first version of this extremely insightful and thought provoking paper. Their amendments are appropriate and welcome and their critical response to my suggestions indicate that there is much more common ground between our views in these matters than differences. I note the helpful additional literature they cited which provides a useful context and further support to the argument they put forward. 

 I am convinced this paper makes an important contribution to the ongoing debate about the impact of non-pharma interventions in combating the COVID outbreak.

 Approved with no reservations.

Is the argument persuasive and supported by appropriate evidence?

Yes

Is the work clearly and cogently presented?

Yes

Is the topic of the essay discussed accurately in the context of the current literature?

Yes

Does the essay contribute to the cultural, historical, social understanding of the field?

Yes

Reviewer Expertise:

Health Services research, Programme Evaluations, Implementation Science

I confirm that I have read this submission and believe that I have an appropriate level of expertise to confirm that it is of an acceptable scientific standard.

# Peer review: Reviewer response for version 1

**DOI:** 10.21956/openreseurope.15727.r29149

**Published:** 2022-07-18

**Authors:** Frans Leeuw

**Affiliations:** 1 Faculty of Law, Maastricht University, Maastricht, The Netherlands

The topic of the study is extremely important: an unparallelled programme of public restrictions, big public finance investments in helping companies, even implementing interventions that even in the year before the first outbreak (2019) were, by the WHO, not considered feasible or important (like lockdowns), mass social protests etc. The realist evaluation approach to this large public challenge has been done carefully, to the point and with a certain distance in perspective. The structure of the paper is excellent. The lessons that are suggested have been underpinned as good as it could be done, but of course time will tell what the results in reality in the future may/ will be.

Is the argument persuasive and supported by appropriate evidence?

Yes

Is the work clearly and cogently presented?

Yes

Is the topic of the essay discussed accurately in the context of the current literature?

Yes

Does the essay contribute to the cultural, historical, social understanding of the field?

Yes

Reviewer Expertise:

evaluation research, behavioral and social studies

I confirm that I have read this submission and believe that I have an appropriate level of expertise to confirm that it is of an acceptable scientific standard.

# Response

**Published:** 2022-08-05

**Authors:** Pilar SERRANO-GALLARDO

**Affiliations:** Universidad Autónoma de Madrid, Spain

We would like to thank Dr Leeuw for his review and comments on our work. As Dr Leeuw points out, we hope that the complex lessons we propose can be taken into account in ongoing research on pandemic management across Europe and other regions of the world.

# Peer review: Reviewer response for version 1

**DOI:** 10.21956/openreseurope.15727.r29151

**Published:** 2022-05-23

**Authors:** Axel Kaehne

**Affiliations:** 1 Health Services Research, Edge Hill University, Lancashire, UK

This is a fascinating and deeply thought provoking paper examining the effectiveness of the social control measures during the COVID pandemic. The narrative mainly uses evidence from two countries, the UK and Spain and argues that non-pharmaceutical interventions to reduce COVID infections in the population were largely ineffective. In addition, the authors set out in detail why they think measures such as 'lock-downs' were likely to fail in achieving lower infection rates. They utilise a (loosely defined) realist interpretative framework aligned with the 7 different modes of complexity theory. The theory is clearly laid out and the structure of the paper is also clear detailing their response for each mode in turn.

 Whilst there is generally little in the paper that I would disagree there are a couple of questions that may, if asked, reveal alternative perspectives which, turn may help widen pool of possible explanations again. 

 I will set out some of these below. The authors may find it useful to address/add comments on each of these in the appropriate places their paper.

 1) Methods: the authors utilise a realist review approach (page 5) but there is less clarity on how this was done in practice. I think it would be useful for the authors to sketch their realist review approach in a brief separate section to allow readers to gain a better understanding of how the conclusions for each mode were reached. 

 2) There are some statements in the paper where findings have perhaps been a bit overinterpreted or interpretative frames of references have shifted imperceptibly. Please consider rephrasing to ensure that initial theses/arguments are not expanded later in the text; example: page 5, paragraph 1: 2) 'control was never achieved...' I think the diagram is not showing that control was never achieved. The Belgium graph clearly shows a different picture and the Austria graph does appear so too. I would suggest to limit the statement to UK and Spain which are also the focus of the subsequent sections (and for which this statement holds true) and reconsider the temporal qualification 'never'.

 3) Some statements appear to make political points rather than contribute to the analysis. On page 7, last paragraph, the authors talk about 'transfer of resources' and its impact on health systems. Whilst I agree with the observation, I am not sure this is relevant within the focus of their paper since the aim is to elucidate the failure of social restrictive measures on infection rates. 

 4) page 11, third paragraph, second column: the authors put forward an argument about the possibly higher effectiveness of micro-circuits of transmission. However, there is little in their discussion of the seven modes that would **not** apply to micro-circuits (localised control measures). I am not convinced that the size/footprint is a significant factor with regard to those features of social control measures they discussed. And if it was I would like to see this emerge from their realist review. Aspects of complexity would in my opinion apply to micro-circuits just as they do for macro-circuits. In addition, I am not sure their argument in the seven modes points much to an increased effectiveness of social control measures in micro-circuits, and there may be plenty of counter-examples if one were to look closely perhaps (for example German Federal States with local regulations)?

 5) The main thrust of the argument is that the modelling provides poor guidance to effective policy making in this field as static modelling is unable to incorporate feedback loops etc. Yet there is also the possibility to improve modelling as well. The authors should consider mentioning this an option too in their recommendations, starting with localised data aligned with localised COVID responses where this happened. This may still possibly reveal some effectiveness of some social control measures.

 Clearly this paper makes an important contribution to the debate and I am looking forward to see a more methodological explanation in future papers perhaps about how we can use realist review in linking policy solutions to complex social interventions such as social restrictions during pandemics.

Is the argument persuasive and supported by appropriate evidence?

Yes

Is the work clearly and cogently presented?

Yes

Is the topic of the essay discussed accurately in the context of the current literature?

Yes

Does the essay contribute to the cultural, historical, social understanding of the field?

Yes

Reviewer Expertise:

Health Services research, Programme Evaluations, Implementation Science

I confirm that I have read this submission and believe that I have an appropriate level of expertise to confirm that it is of an acceptable scientific standard, however I have significant reservations, as outlined above.

# Response

**Published:** 2022-08-05

**Authors:** Pilar SERRANO-GALLARDO

**Affiliations:** Universidad Autónoma de Madrid, Spain

The authors would like to thank Dr Kaehne for the excellence and diligence of their reports; and especially, for spending a considerable amount of their own time in drafting such detailed responses. Dr. Kaehne finds ‘little to disagree with’ in our paper but seeks further clarification on several points. We thus structure our rejoinder around his five queries.

 1. As requested, we have added a new coda to the original draft, containing some further explanation and key references to the methodology of rapid realist review. We regard Dr. Kaehne as a ‘critical friend’ of realist methodology, given that he has produced searching commentaries of some of Pawson’s previous writing (Kaehne, 2018; Kaehne, 2021). He shares our Popperian instincts that all empirical research is fallible, and that truth is only obtained by the systematic cross-examination of rival theories. He is the editor of the prestigious *Journal of Integrated Care* and thus has vast experience in the evaluation of complex interventions. We note, moreover, that interorganizational collaboration in healthcare is ‘complexity epitomised’. Integrated care is thus a topic that is highly conducive and quite familiar with realist review, as represented for instance in papers by Aunger et al. (2021a; 2021b). Having said all this, we disagree with him on the following points.

 2. Kaehne thinks we should be more cautious about the extent of our claims on the failure to control the pandemic by means of ‘lockdown’. We heed the point linguistically and have amended a key point in the text (p. 5) to read that ‘management of the virus was never fully achieved through social controls.’ We continue to maintain, however, that real suppression only occurred under the vaccination programme. It is worth looking more closely at the data which underpins the claim about the limitations of lockdown policy. We reproduced a BBC graph which shows the rolling averages of cases in 16 countries. This provides a convenient bird’s eye view and importantly includes evidence on our case studies of the UK and Spain.

 Note that these graphs are reproduced from a much larger international data set from John Hopkins University. Alas this was far too large to reproduce in the ORE paper and we maintain that close scrutiny of the big picture provides yet more evidence for our pessimism about COVID social controls. It is also worth noting the precise scope of our proposition. Our claim is that incidence rates *fluctuate* despite controls. They are irregular, turbulent, disorderly. We are not claiming that there is one profile, that all countries follow the same pathways, that all time-series have the same contours. So indeed, the Belgium and Austria graphs are not the same as those for the UK and Spain. But neither is the Spanish graph remotely the same as the one for the UK. What they have in common is that they are in flux, progress is unpredictable (more on this in point 5). We completely disagree, therefore, that we should limit the scope of our analytic claims to the UK and Spain. We published in ORE in the expectation that readership from a wide range of European countries would recognise the erratic play of the same policy complexities. But the best expression of the actual scope of our analysis is already in the paper’s conclusion ... ‘in complex, liberal democracies, with diverse and disputatious populations, with instant and endless social interaction, with extensive worldwide interconnectedness, and with dispersed administrative systems, the broad picture is that centralised control systems generated outcomes that were partial, short-lived, and indeterminate’.

 3. We totally agree with Kaehne’s strictures about keeping political points out of scholarly analysis (Pawson, 2013). We therefore plead ‘not guilty’ to trading in ‘cheap shots’. We see nothing contentious about the claim that the pandemic often involved the transfer of resources from other healthcare provision. We even note that ‘it is not a given’.

 4. Kaehne doubts the veracity of our suggestion that pandemic social control measures might be improved if informed by local knowledge of how people react to controls; and implemented by people who are respected in those communities. Once again, we are hardly the first to make this suggestion about the significance of *local public health*. See the Lancet editorial (2017) and associated papers for important details on how such community interventions have been successfully implemented. Kaehne is obviously right to maintain that such a change of scale is no panacea and that local systems have their own dogged complexities. We are thus content that our paper makes a mere first step in this thesis, namely that most of the COVID control measures consisted of top-down commands that often went unheard, unheeded, and ignored at the local level. Nowadays, people and communities absorb information in completely novel ways. What goes on in these micro-circuits determines the actual level of compliance.

 5. Our paper is critical of the use of epidemiological models in predicting the likely advance of the pandemic. We argue that despite its significant presence in key decision centres, mathematical biology provides a poor guide to effective policy making. More recently, other authors have extended the same argument (Corteel, 2022; Greenhalgh and Engebretsen, 2022 ). Kaehne suggests that our conclusions should be moderated, including the possibility that modelling itself could be improved by incorporating ideas from complexity theory on feedback loops, non-linearity, and so on. This debate takes us back to the famous quotation attributed the most famous statistical modeller, George Box: "All models are wrong, but some are useful". Kaehne and I debated this some time ago (Pawson, 2021; Kaehne, 2021). Any influence shaping the contours of a disease can *potentially* be included as a parameter in a simulation model. With Box we can thus say that it is impossible to predict every possible influence in pandemic systems, but ‘useful’ models are the ones which capture an optimal mix of causal influences. The problem here is that this optimal mix cannot be known in advance. Complex systems are essentially unpredictable, they are composed of and defined by unacknowledged conditions and unintended consequences (Livesay, 1989). Crisis policies are made without knowledge of the full set of circumstances that created the said crisis, and the ensuing policies go onto create conditions that are unintended by their creators.

 To be sure, the presence and significance of the unacknowledged and the unintended can be researched, but alas – only *retrospectively*. It is useful to pursue this argument around a specific example. Hypothetically, it would be possible to include a parameter in a simulation model that predicted the ‘free-rider problem’, and projected the potential negative impact on public opinion, and on rule-following of the activities of prominent rule-breakers. But how would one ‘estimate’ this influence? In the UK case, there is sound evidence cited in the paper that the activities of one such free rider, Dominic Cummings, a prominent government advisor, had a significant negative impact on compliance. Why? It is a long story, but one might summarise it retrospectively by saying that he was a Machiavellian character with undue influence on the PM, who made prepositions public excuses for a preposterously long private car journey when ‘lockdown’ was at its height. The devil was all in the detail. Another free-rider may get off scot-free. A multitude of capricious, sporadic ‘events’ of this ilk shaped the dwindling support for the government restrictions. But it would take a soothsayer rather than a modeller to foresee them and estimate their impact. Hundreds of predictive models were produced throughout the world during the pandemic.  We know, thanks to Box, that they were all ‘wrong’. But it remains important to test the thesis that they actually proved ‘useful’ in managing the response (though this task is, of course, a question that is way beyond the scope of our review). To what extent did modellers sit around the decision-making tables? To what extent did models feed into policy? To what extent did they improve policy? These are vital questions for further research (and the next pandemic).

 We conclude by thanking Dr Kaehne for their wise words. Debate on all of these matters will not be settled in a single exchange. Public inquires on the handling of the pandemic are about to be launched across Europe. Let us hope that they are prepared to learn *complex* lessons.

**Additional references.**

 Aunger, J. R. Millar, and J. Greenhalgh. (2021a). When trust, confidence, and faith collide: refining a realist theory of how and why inter-organisational collaborations in healthcare work. *BMC Health Services Research*. 21:602. https://doi.org/10.1186/s12913-021-06630-x  

 Aunger, J., R. Millar, J. Greenhalgh, R. Mannion, A. Raffety and H. McLeod. (2021b). Why do some inter-organisational collaborations in healthcare work when others do not? A realist review. *Systematic Reviews.* 10:8 https://doi.org/10.1186/s13643-021-01630-8  

 Corteel, M. (2022). Les boîtes noires de la pandémie: Modélisation épidémiologique et pouvoir politique durant la crise de Covid-19. *Multitudes*. 86:147.-55. https://doi.org/10.3917/mult.086.0147

 Greenhalgh, T. and  E. Engebretsen. (2022). The science-policy relationship in times of crisis: An urgent call for a pragmatist turn. *Social Science and Medicine*, 306:115140. https://doi.org/10.1016/j.socscimed.2022.115140

 Kaehne, A .(2018) What historiography can tell us about evidence-based medicine and policy: A response to Pawson’s ‘The Realist Foundations of Evidence-based Medicine’. *Evaluation. *24(3): 318-24. https://doi.org/10.1177/1356389018781356

 Kaehne, A. (2021) From abstract to ideal–The limits of models. A reply to Pawson’s ‘Boxed in by models’. *Evaluation.* 27 (3): 400-06. https://doi.org/10.1177/13563890211007505 Lancet Editorial. (2017) Public health is local. *The Lancet*.2(9): e387. https://doi.org/10.1016/S2468-2667(17)30170-6

 Livesay, J. (1989) Structuration theory and the unacknowledged conditions of action, *Theory, Culture & Society*, 6(2):263–292. https://doi: 10.1177/026327689006002005

 Pawson, R. (2013) *The Science of Evaluation*. London: Sage.

 Pawson, R. (2021) The coronavirus response: Boxed in by models. *Evaluation.* 27(2):149-67. https://doi.org/10.1177/1356389020968579
